# Thermal and Mechanical Properties of Amorphous Silicon Carbide Thin Films Using the Femtosecond Pump-Probe Technique

**DOI:** 10.3390/ma15062165

**Published:** 2022-03-15

**Authors:** Yun Young Kim

**Affiliations:** School of Mechanical Engineering, Chungnam National University, Daejeon 34134, Korea; y.kim@cnu.ac.kr

**Keywords:** amorphous silicon carbide, thin film, thermal conductivity, Young’s modulus, femtosecond laser ultrasonics

## Abstract

Nanoscale amorphous silicon carbide (a-SiC) thin films are widely used in engineering applications. It is important to obtain accurate information about their material properties because they often differ from those of the bulk state depending on the fabrication technique and process parameters. In this study, the thermal and mechanical properties of a-SiC thin films were evaluated using the femtosecond pump-probe technique, which provides high spatial and temporal resolutions sufficient to measure films that have a thickness of less than 300 nm. a-SiC films were grown using a plasma-enhanced chemical vapor deposition system, and the surface characteristics were analyzed using ellipsometry, atomic force microscopy, and X-ray reflectometry. The results show that the out-of-the-plane thermal conductivity of the films is lower than that of bulk crystalline SiC by two orders of magnitude, but the lower limit is dictated by the minimum thermal conductivity. In addition, a decrease in the mass density resulted in a reduced Young’s modulus by 13.6–78.4% compared to the literature values, implying low Si-C bond density in the microstructures. The scale effect on both thermal conductivity and Young’s modulus was not significant.

## 1. Introduction

Silicon carbide (SiC) thin films are widely used in engineering applications, such as wide-bandgap electronic devices, microelectromechanical systems (MEMS) sensors [1], photovoltaic solar cells [2], light-emitting diodes [3], and hardmask films [4]. In particular, amorphous SiC (a-SiC) has gained attention in the semiconductor industry because its microstructure can be manipulated by adjusting the fabrication methods to achieve different properties tailored to specific applications. It is important to determine the exact properties for designing nanoscale devices, especially in the computer-aided engineering, as the properties of nanoscale materials often differ from those of the macroscopic state and strongly depend on the fabrication conditions, such as the pressure, temperature, types and flow rates of gases, and applied power. The measurement of thin film properties requires proper techniques suitable for the corresponding characteristic length. While conventional testing methods such as the 3-ω method and bulge test can measure the thermal and mechanical properties of thin films, they require sample preparation procedures using a series of MEMS patterning processes. The femtosecond pump-probe technique, however, can more effectively characterize nanoscale materials through quick laser scanning thanks to its high temporal (in the picosecond regime) and spatial (down to a few tens of nanometers in terms of the film thickness) resolutions.

Herein, the out-of-the-plane thermal conductivity and Young’s modulus of a-SiC film were evaluated using the femtosecond laser metrology. A literature review revealed that only a limited number of studies have focused on the thermal and mechanical properties of a-SiC under 500 nm [5,6]. In this study, 100 nm- and 300 nm-thick films were fabricated using a plasma-enhanced chemical vapor deposition (PECVD) system, and their surface characteristics were analyzed using ellipsometry, atomic force microscopy (AFM), and X-ray reflectometry (XRR). The thermal and ultrasonic responses obtained from the pump-probe experiment were analyzed using thermoelastically coupled one-dimensional heat diffusion and wave equations to extract the material properties. Finally, the results were compared to the literature data for validation.

## 2. Materials and Methods

### 2.1. Experimental

a-SiC films were grown on 525 μm-thick 4-inch Si(100) wafers using the PECVD technique (FABstar+ manufactured by Top Technology Ltd., Hwaseong, Republic of Korea). The system was equipped with a 13.56 MHz radio frequency generator, and the applied power was 60 W. The chamber base pressure was 0 mTorr, and the gas flow rates of CH_4_ and SiH_4_ diluted in He to 5% were 500 sccm and 1600 sccm, respectively. The deposition pressure was 1200 mTorr, and the substrate temperature was maintained at 350 °C. The films were fabricated to thicknesses of 100 nm and 300 nm.

**Figure 1:**
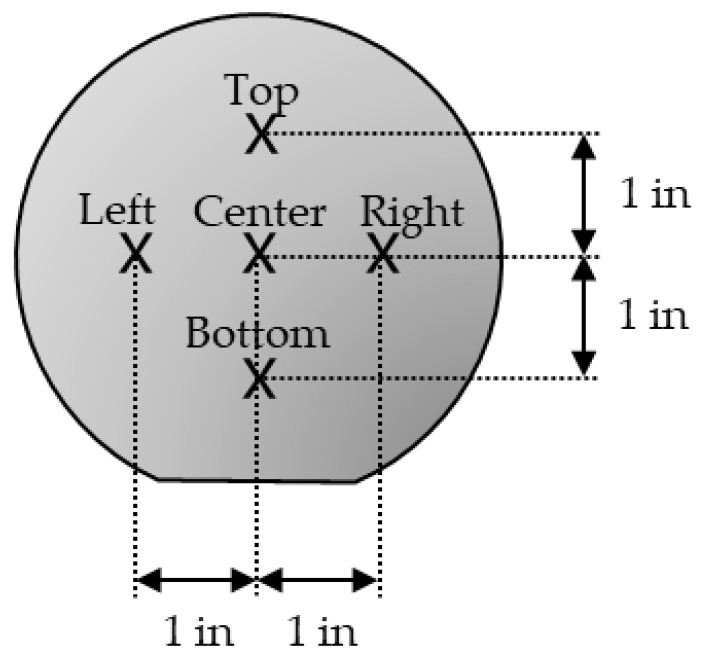
Locations of film thickness measurement.

The film thickness was verified using an ellipsometer (Elli-SE by Ellipso Technology Co., Ltd., Suwon, Republic of Korea). The measurements were performed at five locations on each wafer surface, as shown in Figure 1. The surface morphology over an area of 1 × 1 μm^2^ was imaged using AFM (XE-100 by Park Systems Corporation, Suwon, Republic of Korea) operating in tapping mode. The scan pixels and rate were 256 × 256 and 0.8 Hz, respectively. The film density was evaluated using XRR (X’pert PRO MRD by Malvern Panalytical B.V., Malvern, United Kingdom); Cu-Kα source (wavelength of 1.5406 Å) was used, and the scan range was set from 0° to 1.798° with a step size of 0.002°.

For the laser-optic measurement, an additional layer of 100 nm-thick aluminum (Al) was deposited on the a-SiC surface using an e-beam evaporator (FC-2000 by Ferrotec Corporation, Santa Clara, CA, USA), such that the Al layer could absorb the laser pulses and generate ultrasound in the film. During the deposition, the chamber pressure and the deposition rate were maintained at 1.1 × 10^−7^ Torr and 2.0 Å/s, respectively. At the same time, a bare Si(100) wafer was also installed in the chamber to verify the properties of Al.

The thermal conductivity and Young’s modulus of a-SiC were estimated using the femtosecond pump-probe technique. A Ti:sapphire oscillator (Tsunami by Spectra-Physics, Inc., Milpitas, CA, USA), pumped by a 5 W continuous-wave laser at a wavelength of 532 nm (Millenia Pro by Spectra-Physics, Inc., Milpitas, CA, USA), was used to produce laser pulses of 120 fs width at a repetition rate of 80 MHz; the wavelength was 780 nm. The two-color scheme was employed to enhance the signal-to-noise ratio such that the wavelength of the pump beam was converted to 390 nm using a nonlinear *β*-barium borate crystal. The pump beam was modulated at 1 MHz by an electro-optic modulator (Model 380 by Conoptics Inc., Danbury, CT, USA). The thermal decay signal was measured using an optical receiver (1801-FS by New Focus^TM^, Irvine, CA, USA) and a lock-in amplifier (SR844 by Stanford Research Systems, Sunnyvale, CA, USA). The movement of a delay line, which was a retroreflective mirror mounted on a motorized linear stage, was configured to have time steps of 2.7 ps/pt and 0.5 ps/pt for the thermal and ultrasonic responses, respectively. A detailed schematic of the optical setup is presented elsewhere [7]. Each sample was measured three times at the same spot and the results were averaged.

### 2.2. Theory and Computation

**Figure 2:**
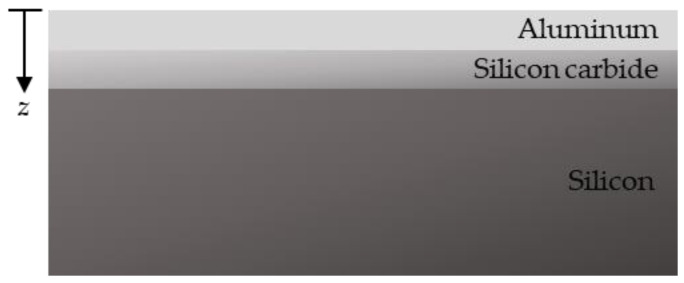
The coordinate system. The silicon substrate is a semi-infinite medium.

The one-dimensional heat diffusion equation was solved to determine the thermal conductivity of a-SiC. The coordinate system is defined as shown in Figure 2. The governing equation is written as follows:

(1) $$\rho C_{p}\frac{\partial T\left(z,t\right)}{\partial t}=\kappa \frac{\partial^{2}T\left(z,t\right)}{\partial z^{2}}+W$$

where *ρ* is the density, *C_p_* is the specific heat, *T* is the temperature, *t* is the time, *κ* is the thermal conductivity, and *z* is the depth. *W* represents the laser heating function given as follows [8]:

(2) $$W\left(z,t\right)=\{\begin{array}{cc} \frac{I_{0}\left(1-R\right)\beta }{2}\exp\left(-\beta z\right)\sin^{2}\left(\frac{\pi t}{2\tau }\right) & 0\leq t\leq 2\tau \\ 0 & t\geq 2\tau ,\;t<0 \end{array}$$

where *I*_0_ is the laser intensity, *R* is the reflectance, *β* = 4π*k*/*λ* is the absorption coefficient, *k* is the extinction coefficient, *λ* is the pump beam wavelength, and *τ* is the laser pulse width. *k* = 4.71 [9] was used and the pump beam was only absorbed in the Al layer.

The boundary conditions are imposed such that the heat conduction on the Al surface (*z* = 0) is negligible due to the low thermal conductivity of air:

(3) $$\kappa_{\mathrm{Al}}\frac{\partial T_{\mathrm{Al}}\left(0,t\right)}{\partial z}=0$$

In addition, the thermal boundary resistance (TBR), *G*, across the Al/a-SiC interface is expressed as follows:

(4) $$-\kappa_{\mathrm{Al}}\frac{\partial T_{\mathrm{Al}}\left(d,t\right)}{\partial z}=\frac{1}{G}\left[T_{\mathrm{Al}}\left(d,t\right)-T_{a\text{-}\mathrm{SiC}}\left(d,t\right)\right]$$

(5) $$-\kappa_{\mathrm{SiC}}\frac{\partial T_{a\text{-}\mathrm{SiC}}\left(d,t\right)}{\partial z}=\frac{1}{G}\left[T_{\mathrm{Al}}\left(d,t\right)-T_{a\text{-}\mathrm{SiC}}\left(d,t\right)\right]$$

where *d* is the thickness of Al. TBR across the a-SiC/Si interface can be expressed similarly.

Equation (1) is a transient problem. The film is initially at rest, and hence, the following initial conditions are applied:

(6) T(z,0)=0

Next, the surface displacement, *u*, is obtained to estimate the Young’s modulus of a-SiC by predicting the longitudinal bulk wave propagation behavior in the film thickness direction. It requires the solution of a thermoelastically coupled wave equation [8]:

(7) $$\rho \frac{\partial^{2}u\left(z,t\right)}{\partial t^{2}}=c\frac{\partial^{2}u\left(z,t\right)}{\partial z^{2}}-B\frac{\partial T\left(z,t\right)}{\partial t}$$

where *c* is the effective elastic modulus, and *B* is the product of *c* and the thermal expansion coefficient. The boundary conditions at the Al/a-SiC interface are obtained from the continuity of displacement and traction:

(8) $$u_{\mathrm{Al}}\left(d,t\right)=u_{a\text{-}\mathrm{SiC}}\left(d,t\right)$$

(9) $$c_{\mathrm{Al}}\frac{\partial u_{\mathrm{Al}}\left(d,t\right)}{\partial t}-B_{\mathrm{Al}}T_{\mathrm{Al}}\left(d,t\right)=c_{a\text{-}\mathrm{SiC}}\frac{\partial u_{a\text{-}\mathrm{SiC}}\left(d,t\right)}{\partial t}-B_{a\text{-}\mathrm{SiC}}T_{a\text{-}\mathrm{SiC}}\left(d,t\right)\;$$

The boundary conditions at the a-SiC/Si interface can be imposed similarly. The initial conditions are as follows:

(10) u(z,0)=0

(11) $$\frac{\partial u\left(z,0\right)}{\partial t}=0$$

The equations were solved using the finite difference method, and the experimental data were curve-fitted using the numerical results to quantify the thermal and mechanical properties.

## 3. Results

### 3.1. Sample Characteristics

Table 1 shows the film thickness data measured using an ellipsometer. The thickness variation was within ±2.6%, and the five-point average values of the 100 nm- and 300 nm-thick films were 98.5 nm and 301.9 nm, respectively. The measurement uncertainty of the ellipsometry is 5%.

**Figure 3:**
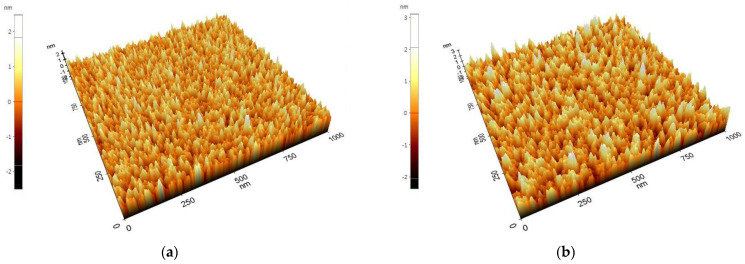
Atomic force microscope images of the a-SiC surfaces: (**a**) root-mean-square roughness (*Rq*) of the 100 nm-thick film was 0.481 nm; (**b**) *Rq* of the 300 nm-thick film was 0.628 nm.

Figure 3 shows the surface morphology of a-SiC obtained from AFM. The root-mean-square roughness of the 100 nm- and 300 nm-thick films were 0.481 nm and 0.628 nm, respectively.

**Figure 4:**
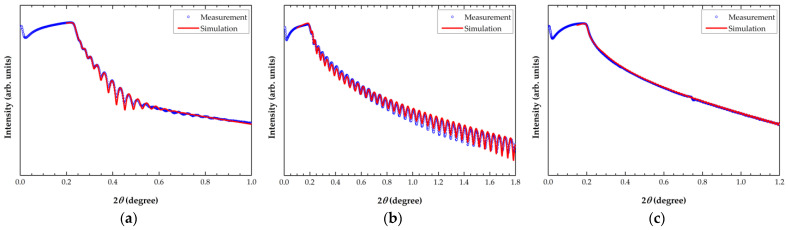
XRR measurement results: (**a**) 100 nm Al, (**b**) 100 nm a-SiC, and (**c**) 300 nm a-SiC.

The XRR measurement results are shown in Figure 4. For the Al film, a mass density of 2.70 g/cm^3^ was obtained for a film thickness of 100.5 nm and roughness of 3.542 nm in the simulation. This density value is practically the same as the literature value for bulk. For the 100 nm- and 300 nm-thick a-SiC films, density values of 1.86 g/cm^3^ and 1.89 g/cm^3^ were obtained, respectively. The film thickness and roughness values used in the simulation were 98.4 nm and 0.552 nm for the 100 nm a-SiC, respectively, and 306.2 nm and 0.743 nm for the 300 nm, respectively.

### 3.2. Femtosecond Pump-Probe Experiment Results

#### 3.2.1. Al Film

**Figure 5:**
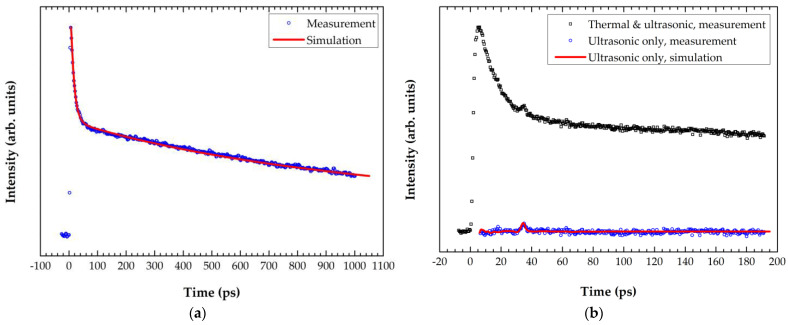
Laser-ultrasonic measurement and curve-fitting results for the 100 nm Al film: (**a**) *κ*_Al_ = 210 ± 10 W/(m·K) and *G*_Al/Si_ = 4.5 ± 0.5 m^2^·K/GW were obtained from the thermal response; (**b**) *E*_Al_ = 56 ± 2 GPa was obtained from the ultrasonic response.

Before the a-SiC measurement, the thermal and ultrasonic responses from the Al film were analyzed to minimize the uncertainty associated with the material properties. Figure 5a shows the thermal decay signal as the optical energy absorbed on the Al surface diffuses into the film. *κ*_Al_ = 210 ± 10 W/(m·K) was obtained from the curve fitting using *ρ*_Al_ = 2.70 g/cm^3^, as verified by the XRR, *C_p_*_,Al_ = 900 J/(kg·K), and *G*_Al/Si_ = 4.5 ± 0.5 m^2^·K/GW. Meanwhile, the properties of Si used in the simulation were *ρ*_Si_ = 2.30 g/cm^3^, *C_p_*_,Si_ = 700 J/(kg·K), and *κ*_Si_ = 148 W/(m·K). *κ*_Al_ of pure bulk Al is typically 237 W/(m·K), but the value is often lower in the form of a thin film. For example, *κ*_Al_ of films with thicknesses ranging from 20 nm to 200 nm varied from 58 ± 30 W/(m·K) to 243 ± 39 W/(m·K), studied using an electrical micropulse generation system [10]. The ultrasonic response of the film is depicted in Figure 5b. To distinguish the echo peak of the longitudinal bulk wave reflected from the Al/Si interface, the thermal response by simulation was subtracted from the measurement data, as represented by the open circles in the plot. The best fit yielded *E*_Al_ = 56 ± 2 GPa for a Poisson’s ratio of 0.34.

#### 3.2.2. a-SiC Films

**Figure 6:**
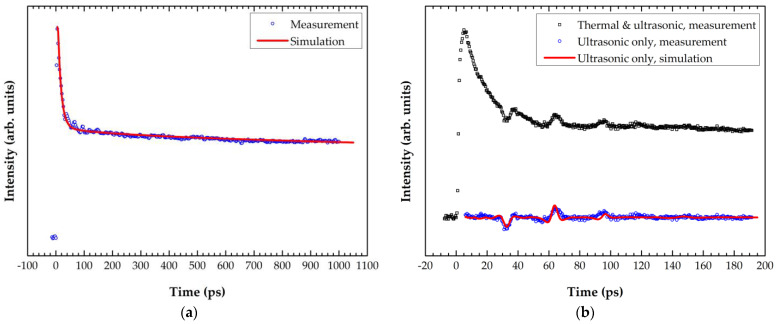
Laser-ultrasonic measurement and curve-fitting results for the 100 nm a-SiC: (**a**) *κ*_a-SiC_ = 1.0 ± 0.2 W/(m·K) and *G*_Al/a-SiC_ = 3.7 ± 1.4 m^2^·K/GW were obtained from the thermal response; (**b**) *E*_a-SiC_ = 67 ± 3 GPa was obtained from the ultrasonic response.

Figure 6 shows the laser-ultrasonic measurement results of the 100 nm a-SiC film. *κ*_a SiC_ = 1.0 ± 0.2 W/(m·K) was obtained using *ρ*_a SiC_ = 1.86 g/cm^3^ and *C_p_*_,a-SiC_ = 680 J/(kg·K), as depicted in Figure 6a. *G*_Al/a-SiC_ was estimated as 3.7 ± 1.4 m^2^·K/GW, whereas *G*_a-SiC/Si_ could not be determined due to the length limit of the delay line. The thermal diffusion length (*Λ*) is given by:

(12) $$\Lambda =\sqrt{Dt}$$

where *D* is the thermal diffusivity ($=\kappa /\rho C_{p}$). Considering the values of *Λ*_Al_ and *Λ*_a-SiC_, a time delay of 12.7 ns would be required for the heat to reach the a-SiC/Si interface; however, it is out of the measurement scope of the present experimental apparatus. Figure 6b shows the longitudinal bulk wave propagation behavior in the 100 nm a-SiC film. Peaks at 32 ns and 64 ns are the echoes from the Al/a-SiC and a-SiC/Si interfaces, respectively. The value of *E*_a-SiC_ = 67 ± 3 GPa was obtained from the curve fitting.

**Figure 7:**
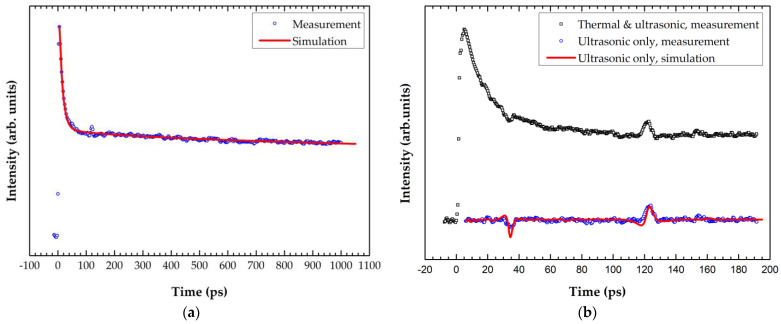
Laser-ultrasonic measurement and curve-fitting results for the 300 nm a-SiC film: (**a**) *κ*_a-SiC_ = 1.1 ± 0.2 W/(m·K) and *G*_Al/a-SiC_ = 5.5 ± 1.6 m^2^·K/GW were obtained from the thermal response; (**b**) *E*_a-SiC_ = 76 ± 3 GPa was obtained from the ultrasonic response.

Figure 7 shows the measurement results of the 300 nm a-SiC films. *κ*_a SiC_ = 1.1 ± 0.2 W/(m·K) and *G*_Al/a-SiC_ = 5.5 ± 1.6 m^2^·K/GW were obtained from the thermal response and *E*_a-SiC_ = 76 ± 3 GPa from the ultrasonic response. Figure 7b shows that the echo peak from the a-SiC/Si interface was delayed to 123 ps due to the increase of the a-SiC layer thickness.

## 4. Discussion

The thermal transport characteristics of amorphous solids are known to be inferior to those of crystalline solids due to more scattering of phonons by irregular microstructures [6]. For example, studies have shown that the thermal conductivity values of bulk crystalline 3C-, 4H-, and 6H-SiC at room temperature are 60 W/(m·K) [11], 104 W/(m·K) [12], and 160 W/(m·K) [13], respectively. In the case of a-SiC films, however, the value drops to a range from 0.83 ± 0.45 W/(m·K) to 1.62 ± 0.17 W/(m·K) [5,6]. The results in this study are consistent with these reported observations. Meanwhile, the lower limit of the thermal conductivity value of an amorphous material is determined by the minimum thermal conductivity theory first proposed by Slack [14] and later modified by Cahill and Pohl [15]:

(13) $$\kappa_{\min}={\left(\frac{\pi }{6}\right)}^{1/3}k_{B}n^{2/3}\underset{i}{\sum }v_{i}{\left(\frac{T}{\theta_{i}}\right)}^{2}\int_{0}^{\theta_{i}/T}\frac{x^{3}e^{x}}{{\left(e^{x}-1\right)}^{2}}dx$$

where *k*_B_ is the Boltzmann constant, *n* is the number density of atoms, *v_i_* is the sound speed of three modes (two transverse and one longitudinal), and *θ_i_* is the cutoff frequency. It is known that *κ*_min_ of a-SiC is 0.95 W/(m·K) [6], which is lower than those of the present results.

The change in the thermal conductivity with a decrease in the film thickness from 300 nm to 100 nm is negligible as the dimension is not comparable to the phonon mean free path *L* determined by *D* and the elastic wave velocity *v* = (*E*/*ρ*)^1/2^ [11]:

(14) L=3D/v

For the 100 nm a-SiC in this study, *L* is estimated as 3.87 Å. A molecular dynamics study [16] showed that the size effect is pronounced as the film thickness increases from 10 to 50 nm, such that *κ* increases from 1.38 W/(m·K) to 1.57 W/(m·K).

Previous research shows that the Young’s moduli of 3C-SiC, 4H-SiC, single-crystal *α*-SiC(001), and single-crystal *β*-SiC(111) are 280–350 GPa [17], 410 GPa [18], 499 ± 2 GPa, and 440 ± 16 GPa [19], respectively. PECVD-grown a-SiC usually possesses a lower Young’s modulus, in the range of 88–310 GPa [4,17,20,21,22,23]. The properties of the films in this study are comparable to the results by Khakani et al. [21] and did not change significantly with the thickness. The elastic modulus is influenced by the microstructural characteristics of the film. Kwon et al. [4] observed that the elastic modulus decreased from 126.3 GPa to 113.2 GPa as the plasma power decreased from 1600 W to 100 W, owing to the decrease in the carbon content of the film. Kaneko et al. [24] also reported that the decrease in the Si-C bond content in the film is linearly proportional to the plasma power density. In addition, it was shown that Young’s modulus could be expressed as a linear function of the mass density, which is also correlated with the Si-C bond density [21]. The XRR results in Figure 4 show that the mass density is 1.86–1.89 g/cm^3^, which is lower than that obtained in the previous studies (2.28–2.85 g/cm^3^) [21,22,23]. The Si-C bond density is influenced by the hydrogen concentration in the film as well as structural defects [20]. To clarify the films’ composition and chemical bond characteristics, in the future, a separate investigation is needed using techniques such as Fourier transform infrared spectroscopy, X-ray photon spectroscopy, and Raman spectroscopy.

## 5. Conclusions

In the present study, the thermal and mechanical properties of a-SiC films were investigated using the femtosecond pump-probe technique. Films with thicknesses of 100 nm and 300 nm were grown using a PECVD system, and the film characteristics were analyzed using ellipsometry, AFM, and XRR. Thermal conductivity and Young’s modulus were measured using the femtosecond pump-probe technique. The results showed that the thermal conductivity values ranged from 1.0 ± 0.2 W/(m·K) to 1.1 ± 0.2 W/(m·K) and followed the minimum thermal conductivity theory. The Young’s modulus values ranged from 67 ± 3 GPa to 76 ± 3 GPa; these values are 13.6–78.4% lower than those obtained in previous studies owing to the low mass density, which indicates low Si-C bond density. Both thermal conductivity and Young’s modulus did not exhibit a significant scale effect.

## Figures and Tables

**Table 1:** The ellipsometry data.

Location	Thickness (nm)	
	100 nm SiC	300 nm SiC
Center	98.4	307.8
Left	97.7	292.6
Right	99.0	306.5
Top	98.1	302.7
Bottom	99.2	300.0
Average	98.5	301.9

## Data Availability

All the data is available within the manuscript.
